# A Randomized Controlled Trial Comparing Wide-Awake Local Anesthesia With No Tourniquet (WALANT) to General Anesthesia in Plating of Distal Radius Fractures With Pain and Anxiety Level Perception

**DOI:** 10.7759/cureus.12876

**Published:** 2021-01-23

**Authors:** Mohd Hazim Abd Hamid, Shalimar Abdullah, Amir Adham Ahmad, Parminder Singh Gill Narin Singh, Elaine Zi Fan Soh, Chian Yong Liu, Jamari Sapuan

**Affiliations:** 1 Orthopaedics and Traumatology, Faculty of Medicine, Universiti Kebangsaan Malaysia, Kuala Lumpur, MYS; 2 Hand and Microsurgery Unit, Department of Orthopaedics, Faculty of Medicine, Universiti Kebangsaan Malaysia, Kuala Lumpur, MYS; 3 Anaesthesia, Faculty of Medicine, Universiti Kebangsaan Malaysia, Kuala Lumpur, MYS

**Keywords:** open reduction and internal fixation, osteosynthesis, volar, locking plate, adrenaline, lignocaine

## Abstract

Introduction: Distal end radius fractures are common fractures commonly treated with an option of open reduction and plating. Traditionally, plating is performed under general anesthesia (GA) or regional block. Recently, a new technique of plating under wide-awake local anesthesia with no tourniquet (WALANT) has been introduced. We aim to compare the preoperative anxiety level, intraoperative pain scores, post-operative pain scores, operating time, blood loss and clinical outcome of distal end radius plating with WALANT versus GA with tourniquet.

Methods: We conducted a randomized controlled study on patients with closed fracture of the distal end of the radius requiring open reduction and plating from January 2019 till April 2020. We recruited 65 patients (33 patients in the WALANT group and 32 patients in the GA group). Randomization was done via block randomization. Data were collected to evaluate preoperative anxiety using the Amsterdam Preoperative Anxiety and Information Scale (APAIS) score, intraoperative pain score during injection (baseline) (V1), 10 minutes after injection (V2), during incision (V3), during gentle manipulation (V4), during aggressive manipulation (V5) and during first drilling of screw (V6), blood loss, duration of surgery and post-operative pain score. Additionally, intraoperative visual analog scale (VAS) score was obtained in the WALANT group. At three weeks, six weeks, three months and six months after operation, the Quick Disabilities of Arm, Shoulder and Hand (QuickDASH) scores and range of motion (ROM) of the wrists were obtained.

Results: The average age in the WALANT group was 47.19 (range, 36-64) years and GA group was 49.48 (range, 38-60) years. The mean APAIS score obtained was 7.78 (WALANT group) and 7.36 (GA group) with no statistical difference. For intraoperative VAS, only during V4 and V5 were the scores 1/10; otherwise at all other phases, the VAS score was 0. The average time for surgery was statistically longer in the WALANT group (61.22 minutes) compared to the GA group (55.33 minutes) (p = 0.003). There was no statistical difference in mean blood loss in both groups. The average post-operative VAS showed statistical significance only at 1 hour and 12 hours post-operation with no statistical difference at 2 and 24 hours post-operation. There was no difference in the post-operative ROM including wrist flexion, extension, supination and pronation for both groups up to six months’ follow-up.

Conclusion: There was no statistically significant difference in terms of preoperative anxiety level, intraoperative and post-operative VAS score, amount of blood loss and clinical outcome in both groups for plating of the distal end radius. However, the operating time was slightly longer in the WALANT group. We conclude that distal radius plating under WALANT has similar outcomes to GA. In centres with limited resources, WALANT offers a safe, reliable and cheaper option, reserving GA time for head, abdominal and thoracic surgery.

## Introduction

A new technique known as wide-awake local anesthesia with no tourniquet (WALANT) in which lidocaine and epinephrine are injected for local anesthesia and vasoconstriction, respectively, has been increasingly used by hand surgeons recently [1-7]. This technique enables surgeries to be performed with the patient fully awake and without a tourniquet, thus allowing the intraoperative assessment of function during surgery. WALANT is mainly used for minor procedures of the hand and wrist such as trigger finger and carpal tunnel release as outpatient procedures [1].

Excellent outcomes have been reported with the WALANT technique, pioneered over the last decade. It offers significant advantages such as a faster operating time, and an ability to visualize functional repair of bone and soft tissue, providing at the same time, adequate tension for tendon repair, eliminating the need for a tourniquet in hand surgery together with its associated complications leading to a faster recovery and lower costs for the healthcare system [3]. Therefore, with the advantages listed above, WALANT provides an excellent alternative to general anesthesia (GA) in the treatment of distal end radius fractures. There is a limited number of studies comparing GA, WALANT, regional block and Bier’s block in distal radius plating [2,8-12].

There is a bimodal distribution of distal radius fractures; the pediatric and elderly populations are at the greatest risk of sustaining this injury. Distal radius fracture is commonly associated with high-energy trauma in young adults or osteoporotic injury in the elderly [2,3]. One of the options for the treatment of distal end radius fracture is plating. Plating of the distal radius fractures is typically performed under general or regional anesthesia and with the aid of a tourniquet to minimize bleeding and allow for better visualization of the surgical field.

GA is associated with a number of complications while requires a series of preoperative investigations. WALANT can provide an alternative form of anesthesia avoiding common complications of GA such as nausea and vomiting and allowing high-risk patients to avoid GA [2,8-12]. Surgery under WALANT should be avoided in patients with a history of lignocaine allergy, anxiety disorder or peripheral vascular disease as it may cause peripheral ischaemia. In many regional hospitals, GA is prioritized for head, neck, abdominal and thoracic surgery and the limbs are usually low on the operating list. Thus, without the need for an anesthetist, the operation can be performed earlier. In facilities with limited resources, costs also can be reduced by eliminating the need for preoperative investigations. Tourniquets are associated with pain and discomfort post-operatively [13,14]. In WALANT surgery, a tourniquet is not applied, thus avoiding possible post-operative pain, discomfort and palsy of the arm.

In our knowledge, there are no previous studies comparing WALANT and regional anesthesia or GA that measure the anxiety levels and intraoperative visual analog scale (VAS) scores especially during the period of manipulation or drilling where pain is anticipated.

The aim of our study is to compare WALANT and GA in distal end radius plating and evaluate any differences in perceived discomfort (intraoperative VAS score), anxiety levels, operating time, blood loss and clinical outcome. For anxiety levels, we utilized the Amsterdam Preoperative Anxiety and Information Scale (APAIS) [15,16]. Patients were followed up at three weeks, six weeks, three months and six months where the Quick Disabilities of Arm, Shoulder and Hand (QuickDASH) score and range of motion (ROM) were recorded.

## Materials and methods

This was a prospective randomized controlled study. The Universiti Kebangsaan Malaysia (UKM) Ethics Committee issued approval UKM PPI/111/8/JEP-2019-764. Patients who fulfilled the inclusion criteria were counselled regarding the study and once they agreed, they were included in the study via block randomization to either the WALANT or GA arm using an online randomization programme Sealed Envelope (Sealed Envelope Ltd., London). Age ranged from 18 to 65 years.

We excluded patients with a diagnosis of peripheral vascular disease, diabetes mellitus, ischemic heart disease, psychiatric illness and a history of allergy to lignocaine. Patients demanding GA were also excluded.

All fractures presenting after three weeks from the date of the initial injury were excluded as the fractures would have nearly united and the surgery would be more difficult. Patients with multiple bone fractures or other injuries requiring surgery were also excluded from the study as this would have interfered with the VAS score, operation time and blood loss.

Patients in both groups underwent preoperative assessment by the anesthetic team to ensure they could receive GA if required. Patients were warded for 24 to 48 hours post-operatively for observation before being discharged. Prior to the operation, APAIS scores were obtained. The APAIS comprises six questions and results in a score ranging from 6 to 30 points [15,16]. Higher APAIS scores are associated with higher levels of anxiety.

It is a known fact that 7 mg/kg of a mixture solution of lidocaine 1% with adrenaline is considered safe for upper extremity surgery [1-3]. A total of 50 mL of normal saline was mixed with 50 mL of lidocaine 2%, and 1 mL of 1:1000 adrenaline solution was added; 10 mL of 8.4% sodium bicarbonate was added to reach a final volume of 40 mL.

**Figure 1:**
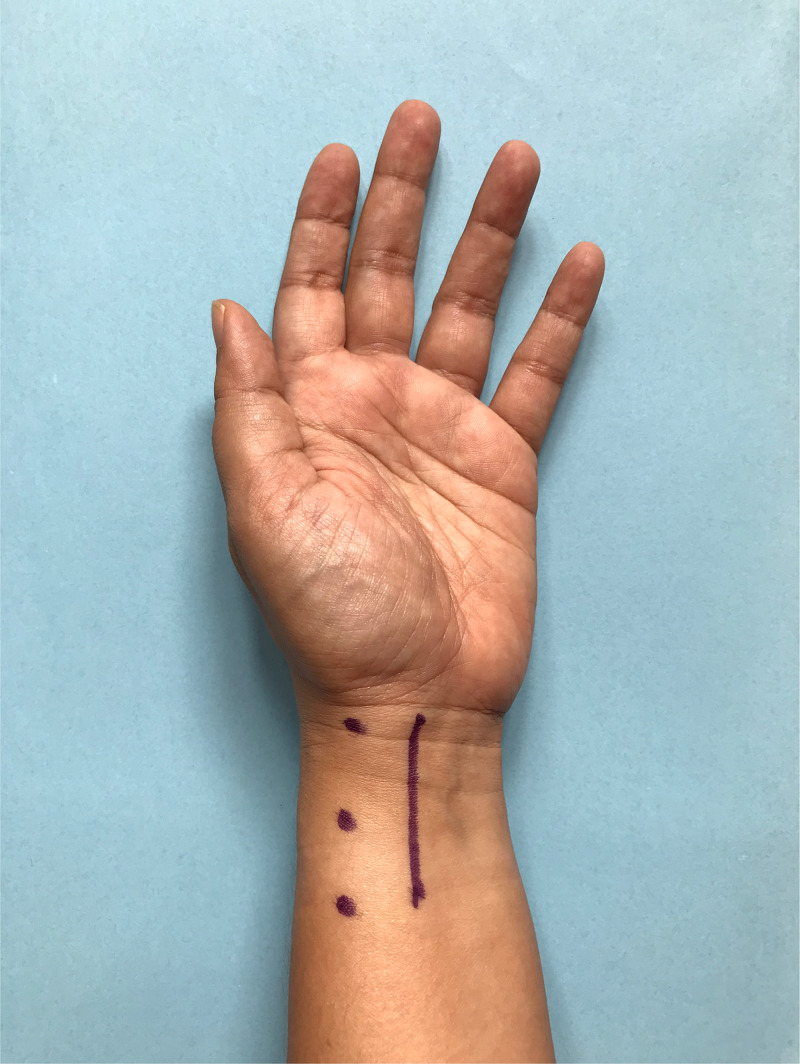
Area of incision The line marked above delineates the area of incision where 10 mL of the solution was injected subcutaneously. A volume of 30 mL was then injected into the periosteal layer at the three dots in the figure - each dot represents an area where 10 mL of the solution was injected.

In the operating theatre, the skin was prepared using the povidone iodine solution. We utilized the method described by Ahmad et al. [8]. First, a total of 10 mL of the local anesthetic was infiltrated subcutaneously using a 27-gauge needle along the modified Henry skin incision (Figure 1).

**Figure 2:**
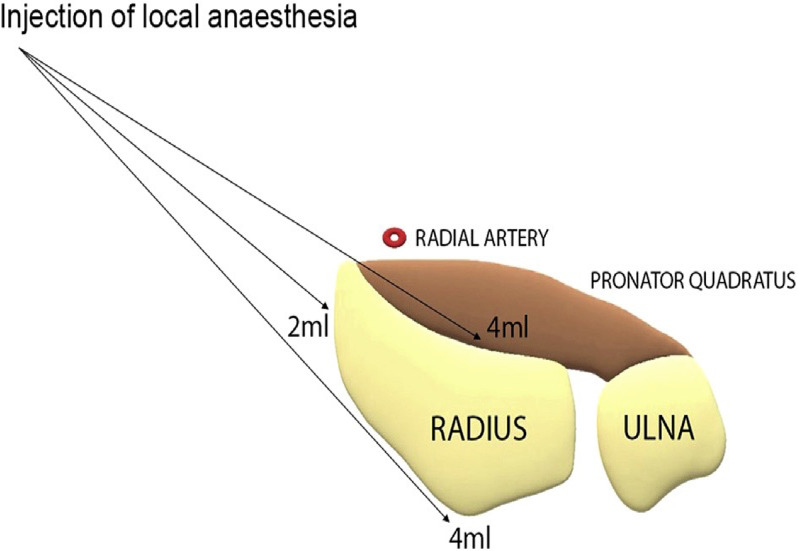
Injection of the local anesthetic WALANT, wide-awake local anesthesia with no tourniquet At each dot, 10 mL of the WALANT solution was injected at different angles into the volar (4 mL), lateral (2 mL) and posterior (4 mL) aspect of the radius within the periosteal layer. Figure reproduced with permission from Ahmad et al. [8].

We ensured that at least 1 cm of visible or palpable subcutaneous local anesthetic was injected proximally and distally to the site of the planned incision. We injected the solution using a 23-gauge needle at the radial border of the radius where it is easily palpable (marked as three dots in Figure 1). Subsequently, another layer of local anesthetic was administered under the periosteal layer to achieve a desired level of anesthesia. This is equivalent to a total of 30 mL of the local anesthetic injected starting proximally with 10 mL at each injection site (Figure 2).

If there was an associated ulnar styloid fracture, an additional volume of 3 mL of the WALANT solution was injected to avoid pain during manipulation. In those cases, the injected volume did not exceed the 43 mL of solution that was within the safe limit of 7 mg/kg for lidocaine with epinephrine. We waited for 30 minutes for the local anesthetic to fully numb the area. It takes an average of 25 minutes for maximal cutaneous vasoconstriction to occur with 1:100,000 epinephrine. The adequacy of anesthesia was assessed by palpating the fracture before the skin incision was performed. Surgery was initiated by orthopedic trainees only if the patient had a pain score of 0. No tourniquet was applied during the surgery. The anesthetist was informed regarding the surgery to be on standby in case a patient required a conversion to GA during the surgery.

VAS scores were recorded intraoperatively in the WALANT group before the injection (baseline) (V1), 10 minutes after the injections (V2), during the incision (V3), during gentle manipulation (V4), during aggressive manipulation (V5) and during the first drilling of screw (V6).

The VAS score was also recorded post-operatively for both WALANT and GA groups at specific intervals until 24 hours post-operatively. Post-operative patients were prescribed with tramadol capsules 50 mg three times a day and paracetamol tablets 1000 mg four times a day. The wound was inspected on the third post-operative day. Sutures were removed at two weeks post surgery. During follow-up, QuickDASH scores and ROM (wrist flexion, extension, pronation and supination) were obtained at three weeks, six weeks, three months and six months. Patients were also referred for physiotherapy for ROM exercise of the wrist and finger flexion.

The QuickDASH consists of 11 items and is employed to evaluate physical function and symptoms in upper limb musculoskeletal disorders (orthotoolkit.com/quickdash/). From the item scores, a summative score is calculated. The final score ranges between 0 (no disability) and 100 (the greatest possible disability). Only one missing item can be tolerated and if two or more items are missing, the score cannot be calculated. The lower the QuickDASH score, the lower the functional disability.

Means and standard deviations were used for standard data tabulation for numerical data. The percentage and frequency were used for categorical data. An independent t-test was used for continuous variables, while a χ^2^ test was used for categorical variables for correlation. A p value of <0.05 was considered significant. All data were analyzed using the statistical application SPSS Statistics, version 26 (IBM Corp, Armonk, NY).

## Results

Demographic details

The demographic information is shown in Table 1.

**Table 1:** Demographic details with AO classification of distal radius fracture patterns WALANT, wide-awake local anesthesia with no tourniquet; GA, general anesthesia

		Frequency	Percentage
Group	WALANT	32	49.2
	GA	33	50.8
Gender	Female	24	36.9
	Male	41	63.1
AO classification	A2	15	23.1
	A3	4	6.2
	B1	5	7.7
	B2	8	12.3
	B3	8	12.3
	C1	5	7.7
	C2	15	23.1
	C3	5	7.7
Ethnicity	Malay	44	67.7
	Chinese	11	16.9
	Indian	10	15.4
	Total	65	100.0

The average age in the WALANT group was 47.19 (range, 36-64) years and the GA group was 49.48 (range, 38-60) years. The majority of the patients were males (63.1%) and 36.9% were females. The majority of the patients were from the Malay race (67.7%, 44), followed by the Chinese (16.9%, 11) and Indians (15.4%, 10). Fractures were classified according to the AO/Orthopaedic Trauma Association classification with the majority of fractures falling into the A2 and C2 types with each group comprising 23.1% (15).

Table 2 shows group makeup and further association.

**Table 2:** Statistical comparison between WALANT and GA groups in terms of gender, fracture classification and ethnicity WALANT, wide-awake local anesthesia with no tourniquet; GA, general anesthesia

Factors	Category	Group		χ^2^	p
		WALANT	GA		
		n (%)	n (%)		
Gender	Female	11 (34.4)	13 (39.4)	0.18	0.675
	Male	21 (65.6)	20 (60.6)		
AO classification	A2	9 (28.1)	6 (18.2)	5.98	0.542
	A3	2 (6.3)	2 (6.1)		
	B1	2 (6.3)	3 (9.1)		
	B2	2 (6.3)	6 (18.2)		
	B3	6 (18.8)	2 (6.1)		
	C1	3 (9.4)	2 (6.1)		
	C2	6 (18.8)	9 (27.3)		
	C3	2 (6.3)	3 (9.1)		
Ethnicity	Malay	20 (62.5)	24 (72.7)	5.03	0.081
	Chinese	4 (12.5)	7 (21.2)		
	Indian	8 (25)	2 (6.1)		

There was an equal gender distribution in both groups with 34.4% (11) females in the WALANT group and 39.4% (13) females in the GA group. Males comprised 65.6% (21) of patients in the WALANT group and 60.6% (20) in the GA group.

Preoperative anxiety scale, duration of surgery and blood loss

**Figure 3:**
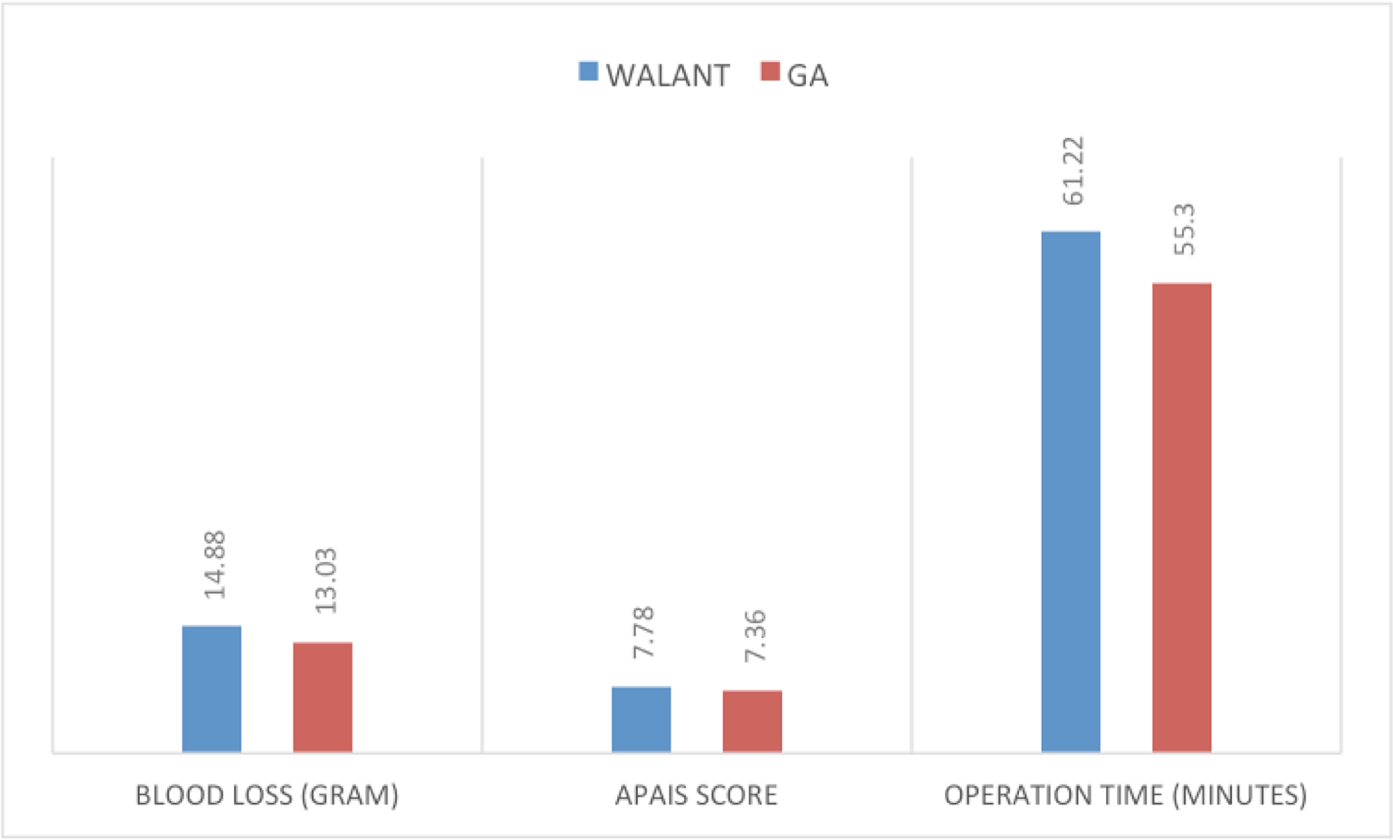
Comparison of blood loss, APAIS score and operative time between WALANT and GA groups APAIS, Amsterdam Preoperative Anxiety and Information Scale; WALANT, wide-awake local anesthesia with no tourniquet; GA, general anesthesia

The mean APAIS score was 7.78 in the WALANT group and 7.36 in the GA group. We found no statistical significance difference between two groups (p = 0.233). APAIS score ranged from 6 to 10 in both groups. The average time for surgery from the incision to the wound closure was 61.22 minutes in the WALANT group and 55.33 minutes in the GA group (p = 0.003). The mean blood loss in the WALANT versus GA group was 14.88 g and 13.03 g, respectively, and we found no statistical difference between the two groups (p = 0.082). Results for the anxiety level before operation, operation time and blood loss are shown in Figure 3.

VAS score intraoperatively and at post-operation

**Figure 4:**
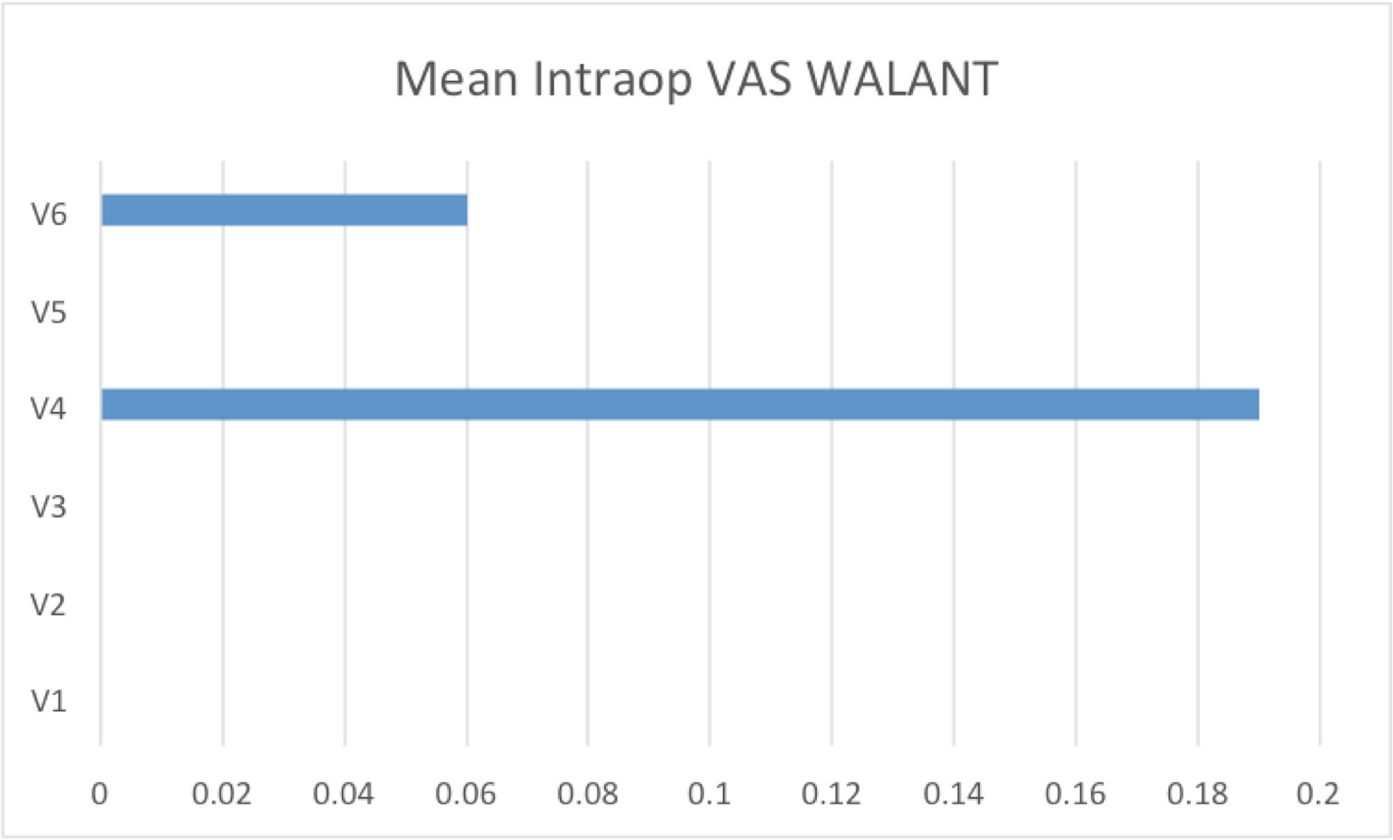
Mean intraoperative VAS score at all six phases of surgery in the WALANT group WALANT, wide-awake local anesthesia with no tourniquet; VAS, visual analog scale

The intraoperative VAS score during plating of distal radius was recorded at V1, V2, V3, V4, V5 and V6 (Figure 4).

During V4 (gentle manipulation), five patients had a VAS score of 1/10. During V5 (aggressive manipulation), three patients had a VAS score of 1/10. No patients had to be converted on table from WALANT to GA.

**Figure 5:**
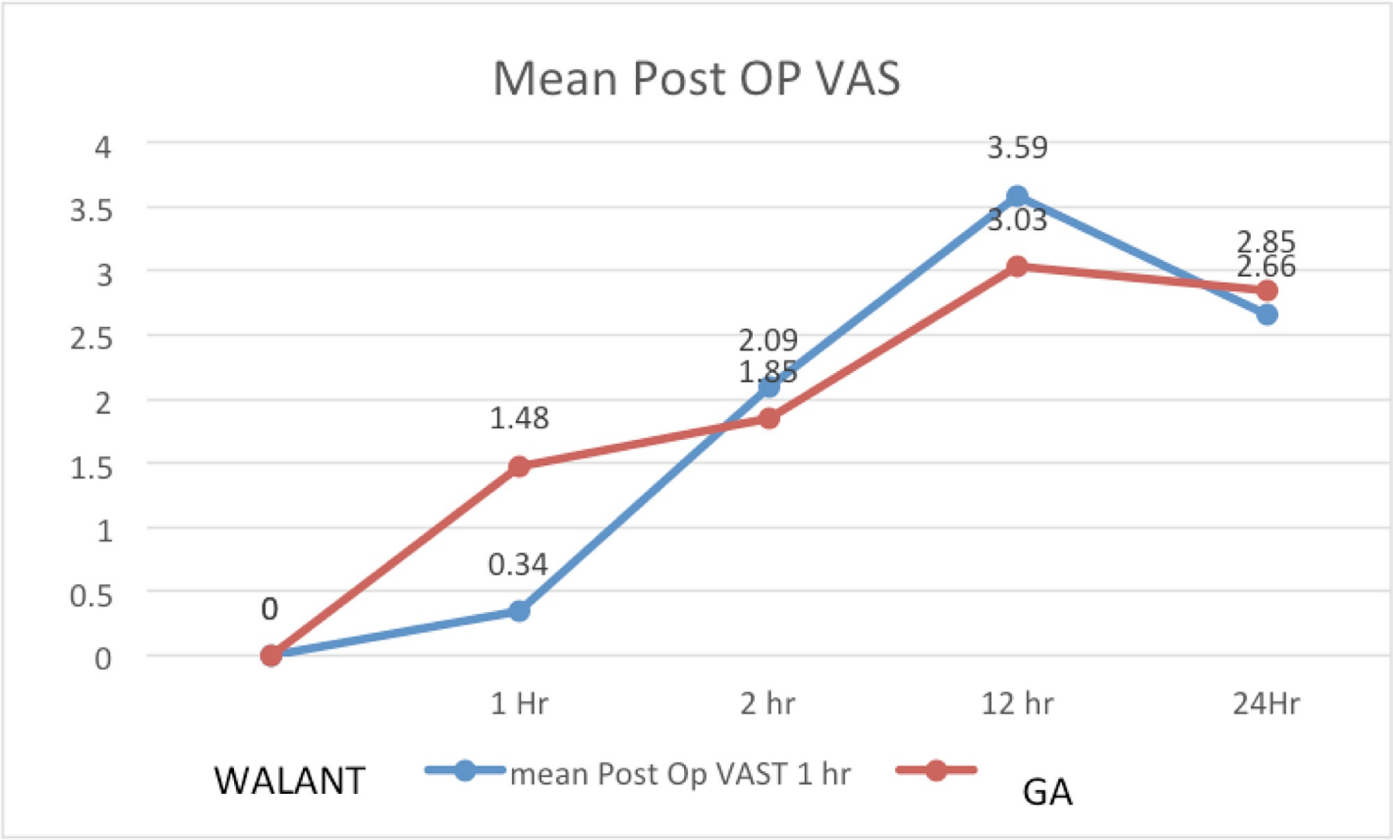
Mean post-operative VAS score at 1 hour, 2 hours, 12 hours and 24 hours in WALANT and GA groups WALANT, wide-awake local anesthesia with no tourniquet; GA, general anesthesia; VAS, visual analog scale

The mean post-operative VAS score at specific time taken for both groups showed statistical significance at 1 hour (p = 0.001) and at 12 hours post-operatively (p = 0.002) (Figure 5). There was no statistically significant difference at 2 (p = 0.213) and 24 (p = 0.277) hours post-operation.

QuickDASH score

**Figure 6:**
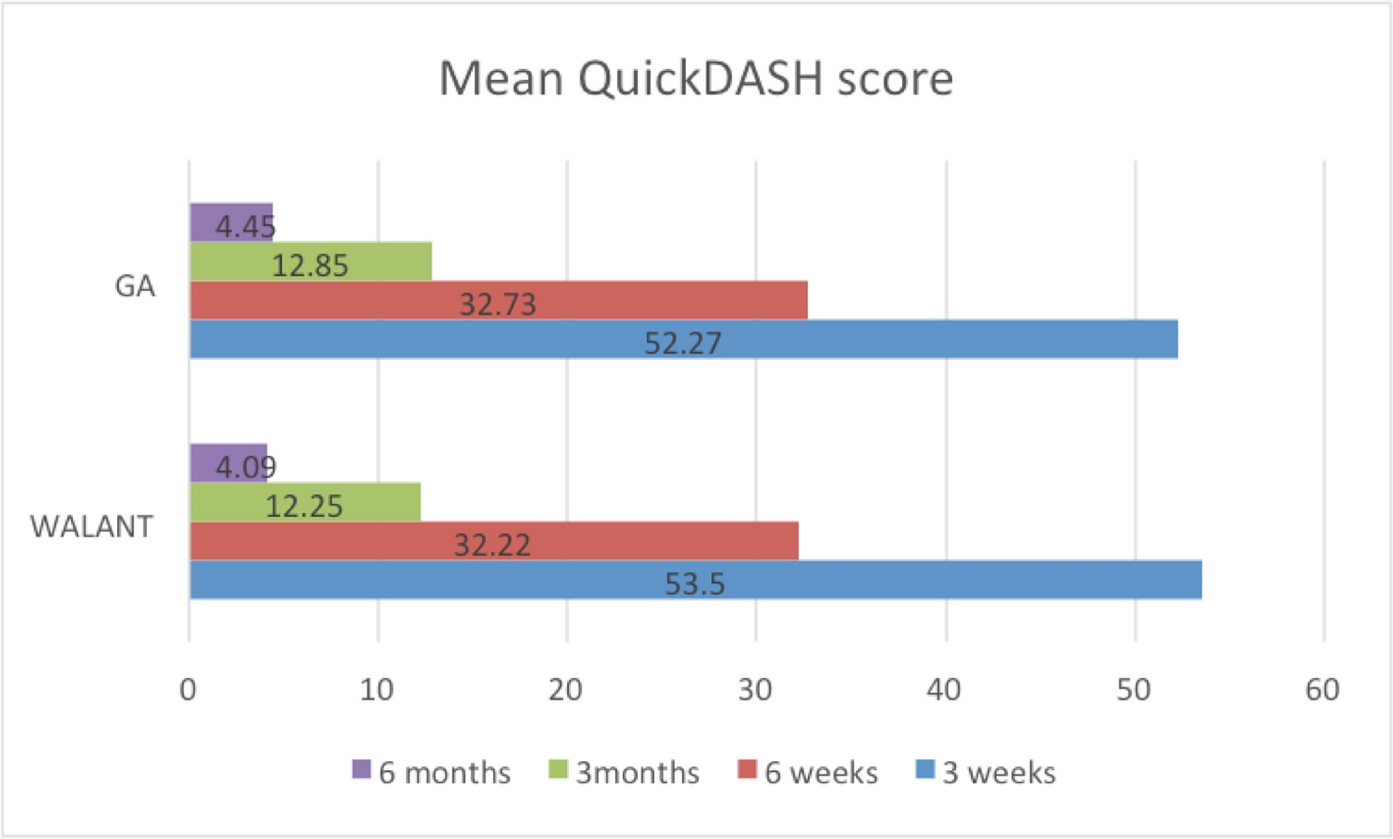
Mean QuickDash score at three weeks, six weeks, three months and six months post-operation between the WALANT and GA groups WALANT, wide-awake local anesthesia with no tourniquet; GA, general anesthesia; QuickDash, Quick Disabilities of Arm, Shoulder and Hand

Post-operative QuickDASH scores were obtained during follow-up at three weeks, six weeks, three months and six months (Figure 6). Both WALANT and GA groups had a reduction in the mean QuickDASH score, from 52.27 at three weeks to 4.45 in the GA group. In the WALANT group, the mean QuickDASH score was reduced from 53.5 at three weeks to 4.09 post-surgery.

Range of motion of the wrist

**Figure 7:**
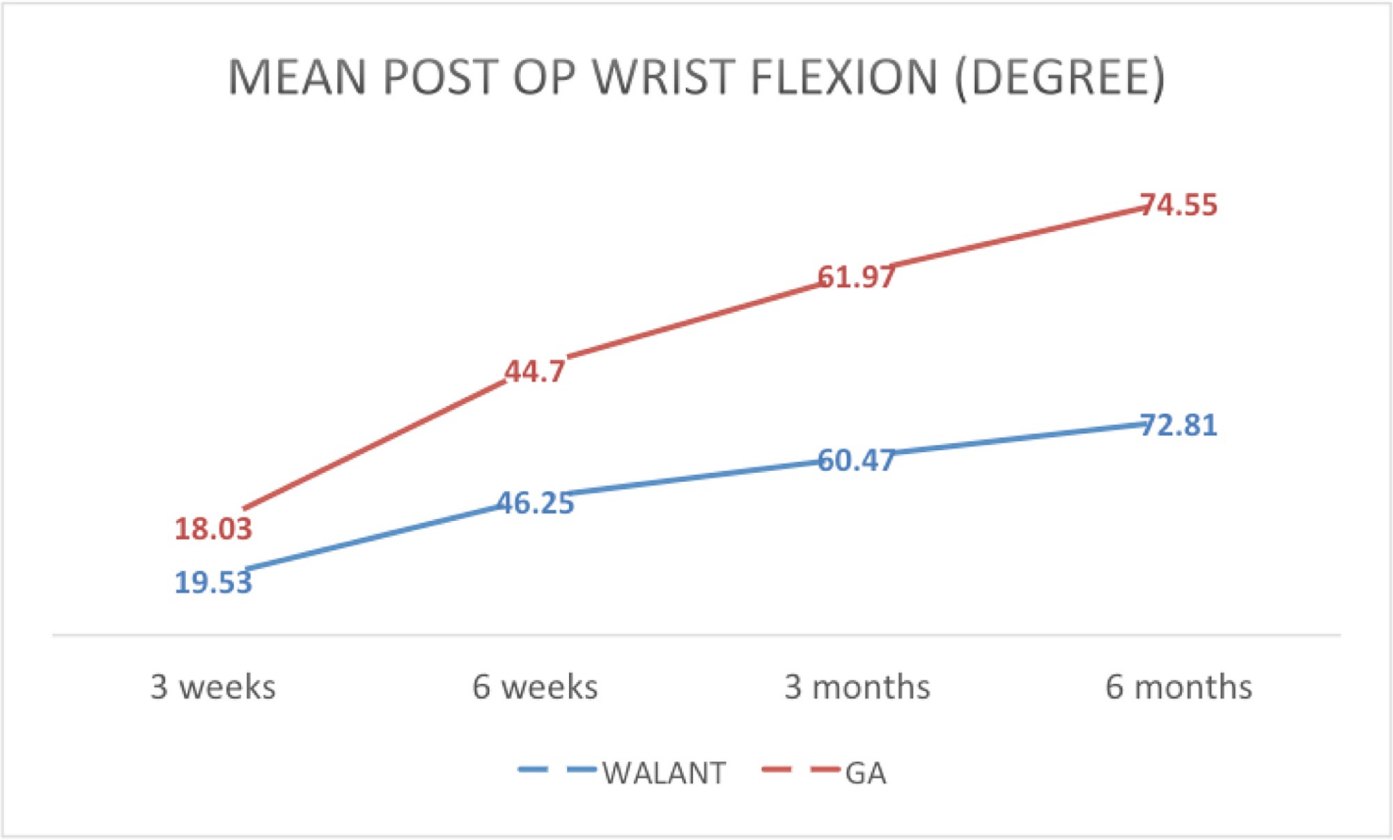
Mean post-operative wrist flexion range of motion at three weeks, six weeks, three months and six months WALANT, wide-awake local anesthesia with no tourniquet; GA, general anesthesia

In this study, the average post-operative wrist flexion for the WALANT group at three weeks, six weeks, three months and six months was 19.53°, 46.45°, 60.47° and 72.81°, respectively. In the GA group, the average wrist flexion was 18.03°, 44.7°, 61.97° and 74.55°, respectively (Figure 7). Both groups showed an improvement in the ROM, but there was no statistically significant difference.

**Figure 8:**
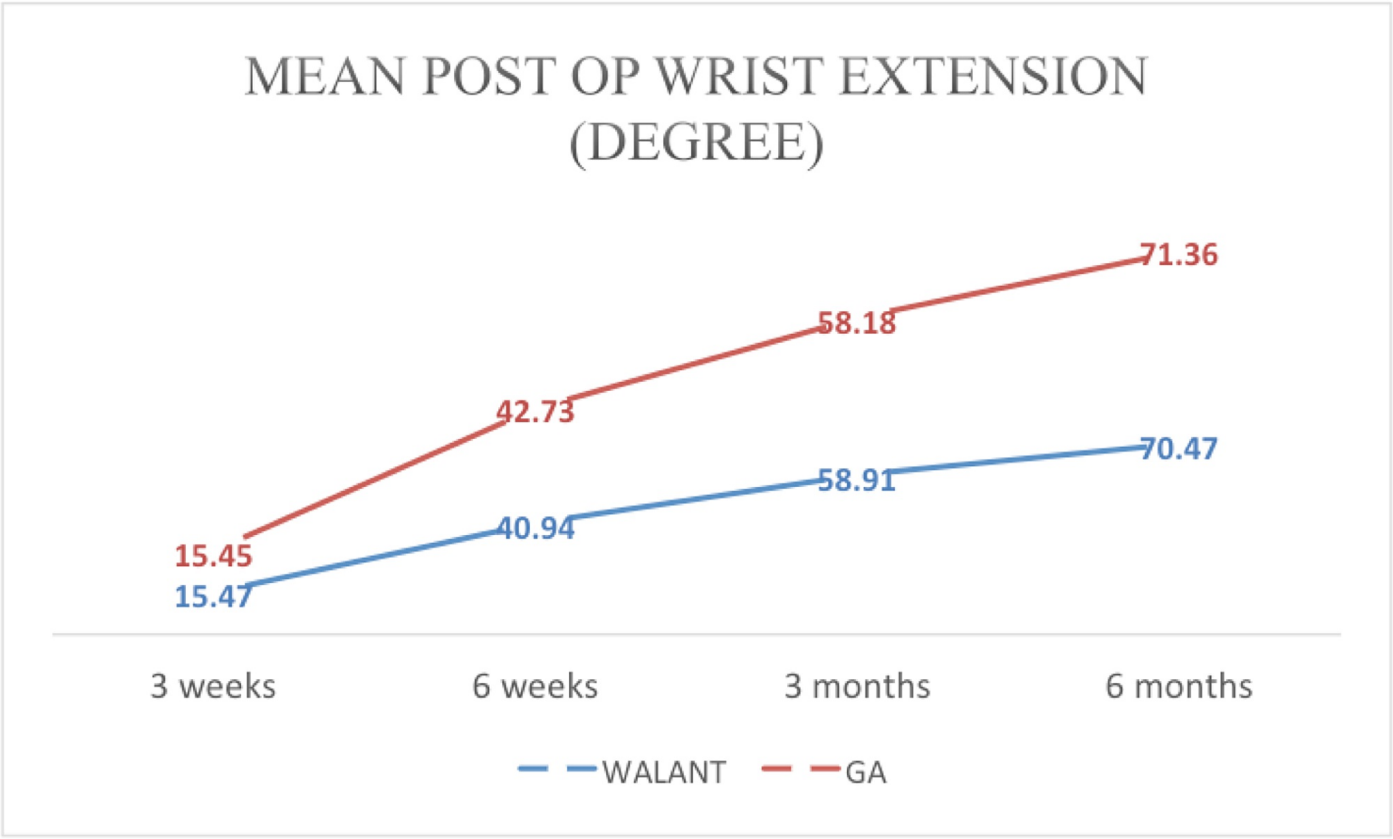
Mean post-operative wrist extension range of motion at three weeks, six weeks, three months and six months WALANT, wide-awake local anesthesia with no tourniquet; GA, general anesthesia

The average post-operative wrist extension at three weeks, six weeks, three months and six months for the WALANT group was 15.47°, 40.94°, 58.91° and 70.47°, respectively. For the GA group, the values were 15.45°, 42.73°, 58.18° and 71.36°, respectively (Figure 8).

**Figure 9:**
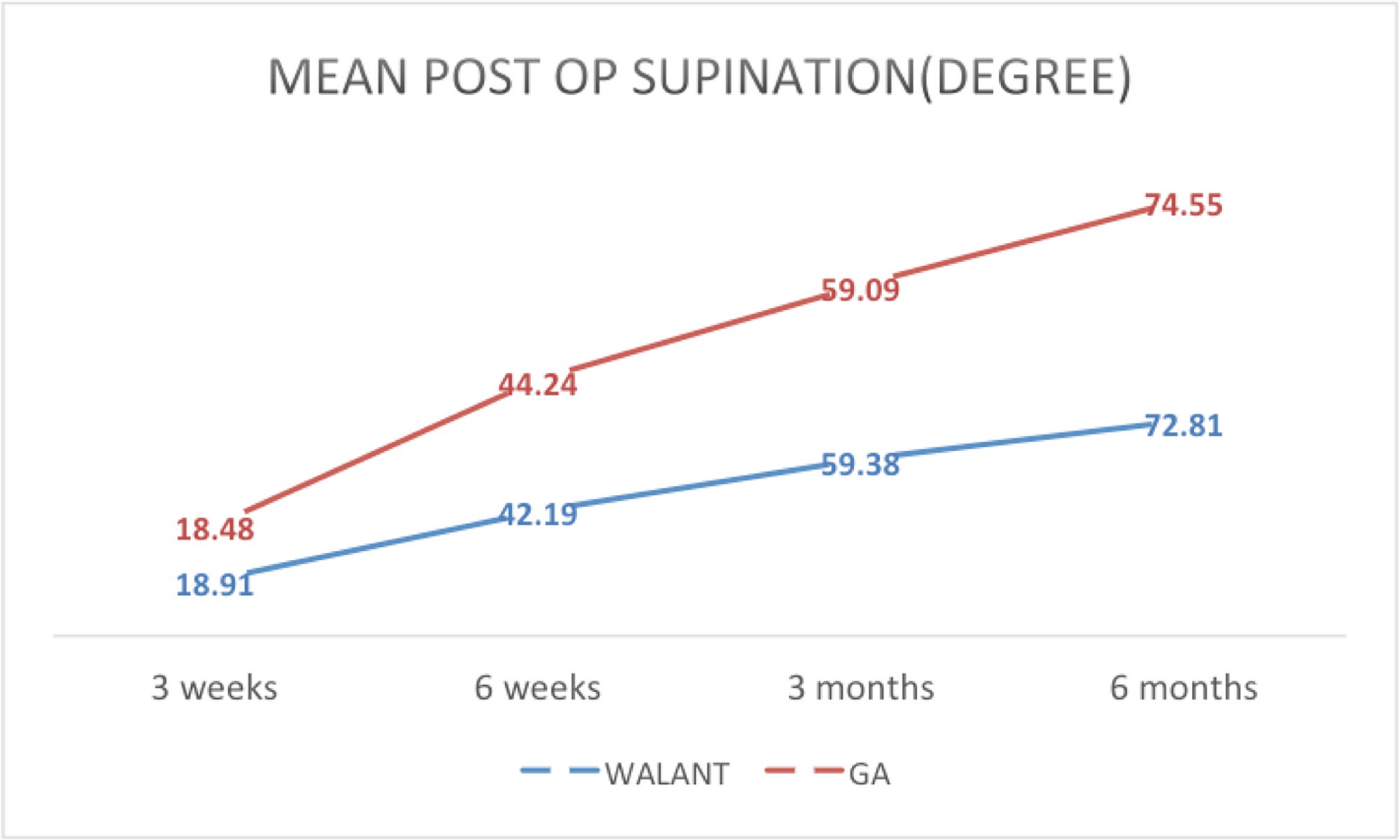
Mean post-operative forearm supination range of motion at three weeks, six weeks, three months and six months WALANT, wide-awake local anesthesia with no tourniquet; GA, general anesthesia

The average supination recorded during follow-up at three weeks, six weeks, three months and six months for the WALANT group was 18.91°, 42.19°, 59.38° and 72.81°, respectively. In the GA group, the values were 18.48°, 44.24°, 59.09° and 74.55°, respectively (Figure 9).

**Figure 10:**
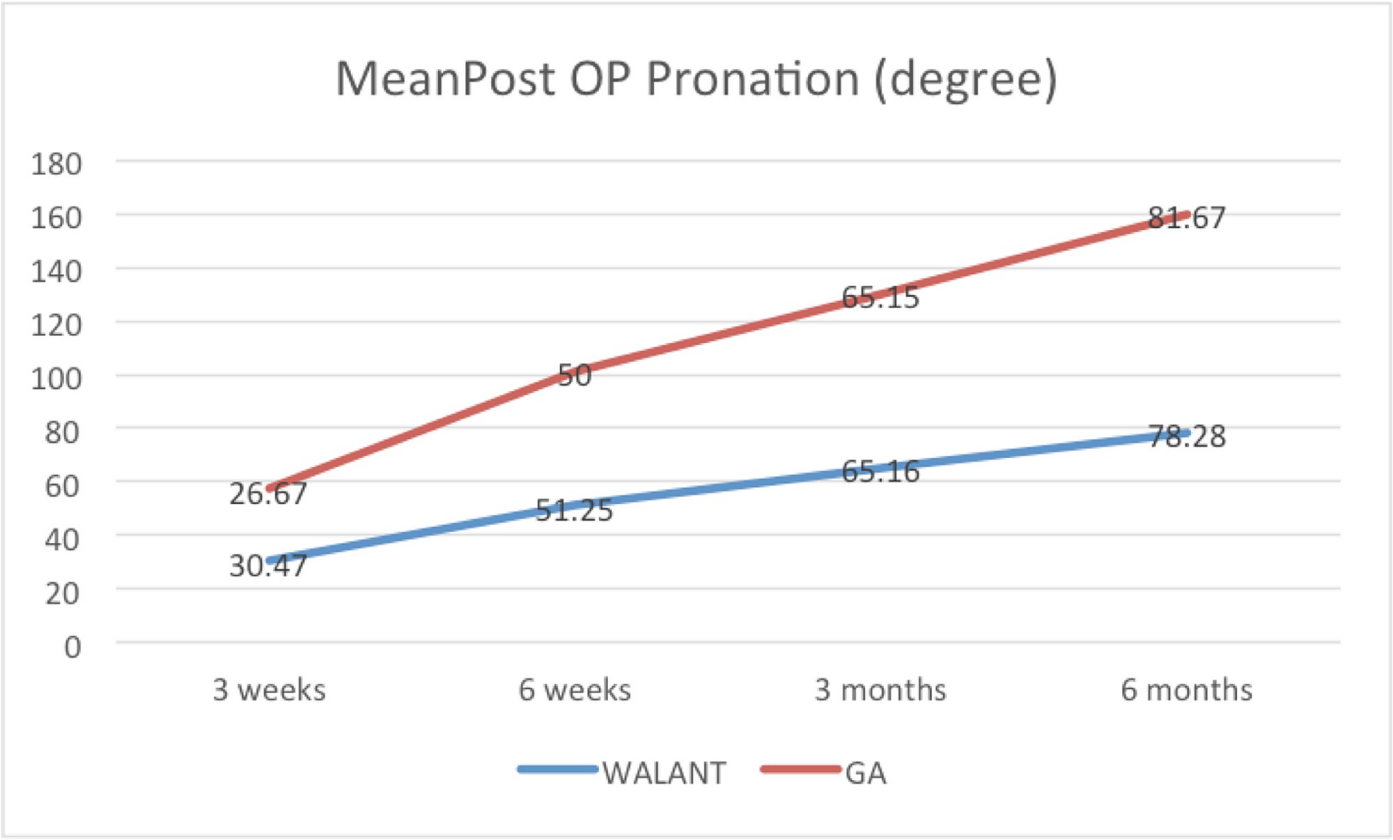
Mean post-operative forearm pronation range of motion at three weeks, six weeks, three months and six months WALANT, wide-awake local anesthesia with no tourniquet; GA, general anesthesia

Pronation exhibited a marked improvement during follow-up for both groups. Pronation was 30.47° at three weeks and 78.28° at six months in the WALANT group. For the GA group, the mean pronation was 26.67° at three weeks and increased to 81.67° at six months from the operation (Figure 10). However, the differences were not statistically significant (p > 0.05).

Adverse reactions

In this study, two revision surgeries were performed and they involved patients from the GA group. One patient required revision surgery due to screw penetration of the wrist joint while another patient underwent a second surgery for distal radio-ulnar joint dissociation requiring pinning. No other complications, such as surgical site infections, finger necrosis, non-union or implant failure, were recorded.

## Discussion

Distal radius fractures involve all age groups. Some need to be managed with open reduction and fixation while others can be adequately treated with cast immobilization. Plating of the distal radius allows early ROM exercises and thus early rehabilitation. Plating of the distal radius is traditionally performed under GA with a tourniquet to reduce bleeding. WALANT is usually employed in soft tissue procedures such as excision of a ganglion cyst or for a carpal tunnel release that do not require a tourniquet. Recently, WALANT has been utilised as an alternative to GA for bony fixation especially of the distal end [2,8-12]. In this study, we attempted to compare the differences in preoperative anxiety, the operation time, the blood loss, the functional outcome using the QuickDASH score and the wrist ROM after distal radius plating via WALANT or GA.

APAIS is commonly used to measure patient anxiety [15]. The average APAIS score was 7.78 in the WALANT group and 7.36 in the GA group. There was no statistically significant difference between the two groups. A total score of 11 or more indicates high anxiety levels [16]. Therefore, WALANT may be contradicted if the APAIS score is 11 or higher.

The average blood loss was 14.8 g in the WALANT group and 13.03 g in the GA group. Both were lower than the average amount of blood loss reported in a study by Huang et al. [2]. In contrast, Tahir et al.’s study reported a much higher amount of blood loss at a mean average of 23.4 mL in the WALANT group compared to GA and Bier’s block groups [11].

The average operative time was longer for the WALANT group (61.22 min) compared to the GA group (55.3 min) and the difference was statistically significant (p = 0.003). Previous studies by Dukan et al. and Tahir et al. found no difference in the operative time between WALANT and GA groups [10,11]. Both GA (n = 14) and WALANT (n = 11) groups had similar AO C2 type of radius fracture. This was to ensure that a complex fracture configuration did not result in a longer operative time.

Intraoperative VAS during the WALANT procedure obtained at specific intervals, namely V1,V2, V3, V4, V5 and V6, showed the VAS score was 0 at all intervals except during aggressive manipulation and screw drilling. However, the VAS score was only 1 in these two periods. We did not need to add any more WALANT solution. Both studies by Dukan et al. and Yi et al. had a few patients with mild pain (ranging from VAS 1 to 4) with pain worsening upon aggressive manipulation and drilling of bone [10,12]. Two patients required additional local anesthesia (WALANT) in Dukan’s study [10] and four patients in Yi et al.’s study [12]. The Tahir et al. study reported a mean highest intraoperative VAS score at 3.5 and yet, this was still lower than the 4.4 score of their Bier’s block group [11].

None of the patients in the WALANT group required conversion to general GA in contrast to Tahir et al.’s study where two patients were converted to GA [11].

Post-operatively, patients were prescribed paracetamol tablets of 1 g three times a day and tramadol tablets of 50 mg three times a day. The average pain score post-operatively at 1 hour, 2 hours, 12 hours and 24 hours for both groups was 4 or less. The average pain score post-operatively was about 1.6 in Huang et al.’s study [2] , but this score was only during the first post-operative day. Patients from Tahir et al.’s study received oral tramadol 37.5 mg/325 mg acetaminophen combination tablets two times a day and had a mean VAS pain score of 1.2 at 24 hours post-surgery compared to 3.0 for the GA and 2.2 for the Bier’s block group [11]. Dukan et al.’s study reported similar pain scores for the WALANT and the regional anesthesia groups [10].

The functional outcome in our study was assessed using the QuickDASH score and the range of motion during follow-up at three weeks, six weeks, three months and six months post-operatively. The QuickDASH score was markedly reduced from three weeks to six months post-operatively for both the WALANT and GA groups, which proved that the type of anesthesia does not affect the functional outcome of the radius fracture fixation.

The average range of motion of the wrist (flexion, extension, supination and pronation) recorded at three weeks, six weeks, three months and six months also showed a significant improvement similar to Dukan’s study that reported better wrist range of motion and QuickDASH scores and an earlier return to work [10]. In contrast, the clinical outcomes in Tahir et al.’s study were not affected by the type of anesthesia [11]. Huang et al. only recorded the degree of flexion and extension of the wrist at one year post-operatively [2].

They are a few limitations to our study: first, our sample size could have been larger and with a longer follow up as other complications could have been detected. Other methods of assessing the functional outcome such as hand grip and pinch strength using a dynamometer could have been utilized.

## Conclusions

Our study showed no statistically significant differences for both groups in terms of anxiety scores, intraoperative or post-operative VAS, blood loss, QuickDASH score and range of motion. The operating time in the WALANT group was slightly longer compared to the GA group. In centres with limited facilities and resources, WALANT offers a safe, reliable and cheaper option, reserving GA time for more complex surgeries such as head, neck and abdominal surgeries.
